# Multiplex One-Step qPCR/RT-qPCR Assays for Detection of Ectromelia Virus, Murine Hepatitis Virus, Reovirus Type 3, and Parvoviruses

**DOI:** 10.3390/vetsci13030217

**Published:** 2026-02-25

**Authors:** Wenxin Luo, Xia Li, Yuewei Zhang, Jianyu Chang, Guoheng Xu

**Affiliations:** 1 College of Veterinary Medicine, China Agricultural University, Beijing 100193, China; 19927531867@163.com (W.L.); monkey830524@163.com (Y.Z.); changjianyu@cau.edu.cn (J.C.); 2 Department of Laboratory Animal Science, Peking University, Beijing 100191, China; xiali@bjmu.edu.cn

**Keywords:** murine hepatitis virus, reovirus type 3, mice minute virus, Ectromelia virus, multiplex real-time PCR (mrt-PCR), molecular detection

## Abstract

The use of experimental animals with unified quality standards is an important condition for ensuring the effectiveness of scientific research. Ectromelia virus (ECTV), murine hepatitis virus (MHV), reovirus type 3 (Reo-3), and murine parvoviruses (MUV) are the four pathogens that need to be eliminated from SPF (Specific Pathogen-Free) level mice. These four pathogens present fast transmission and high pathogenicity, making it difficult to control. The previously described detection methods present substantial limitations in efficiency and accuracy. Thus, there is an urgent need for rapid and precise diagnostic methods to improve prevention and diagnosis efforts. In this study, we developed a one-step multiplex real-time PCR (mrt-PCR) detection method that can simultaneously detect four key viral pathogens causing diseases in laboratory mice without cross-reactivity with other mouse susceptible pathogens. We tested 128 suspected diseased mouse tissue samples collected from Beijing, and the results showed that this new method has higher sensitivity and specificity than ordinary PCR. The detection limit for ECTV, MHV, and MUV was determined to be 1.08 × 10^1^ copies/μL, 1.14 × 10^1^ copies/μL, 2.38 ×10^1^ copies/μL, and 1.08 × 10^1^ copies/μL, respectively. In addition, the assay showed excellent reproducibility, with a coefficient of variation below 1.5%, strong linear correlation (R^2^ > 0.996), and amplification efficiency between 90% and 100%. In summary, the mrt-PCR serves not only as a rapid and accurate detection and early prevention method for laboratory mice but also constitutes a robust tool for microbial quality control in laboratory mice.

## Simple Summary

Laboratory mice are fundamental to biomedical research; however, their health status has a critical influence on experimental outcomes. Ectromelia virus (ECTV), murine hepatitis virus (MHV), reovirus type 3 (Reo-3), and murine parvoviruses (MUV) are four key pathogens requiring exclusion from specific pathogen-free (SPF) mouse colonies to ensure data reliability. This study developed a novel one-step multiplex real-time PCR (mrt-PCR) assay for simultaneous detection of these viruses, optimizing primer and probe concentrations via response surface methodology to achieve balanced amplification efficiency. The assay demonstrated high specificity (no cross-reactivity with other murine pathogens), exceptional sensitivity (detection limits of 1.08 × 10^1^–2.38 × 10^1^ copies/μL), and excellent reproducibility (coefficients of variation < 2%). When applied to 128 clinical mouse tissue samples, it significantly outperformed conventional PCR (e.g., detecting 21.88% vs. 10.16% ECTV positives), proving superior for identifying low-level infections. This mrt-PCR method provides a robust and efficient tool for microbial quality control and early infection prevention in laboratory mouse facilities, thereby safeguarding the validity of animal model-based research.

## 1. Introduction

Animal experiments play an important role in biomedical research [1]. Mice are the most widely used experimental animals for spinal research, and more than half of the biomedical research published in the EU’s report on the use of experimental animals uses mice. This may be due to the sharing of the physiological processes between humans and mice. The mouse genome sequencing project has been completed, and the technology for genome modification is mature. In addition, mice have a short reproductive cycle, strong fertility, and can quickly obtain offspring [2]. It is clear that pathogenic agents causing overt disease in mice represent a serious hazard to research results in studies [3]. Thus, characterization of the health status and microbiological monitoring of the animals in experiments are particularly important.

Ectromelia virus (ECTV), murine hepatitis virus (MHV), reovirus type 3 (Reo-3), and murine parvoviruses (MUV) are four important pathogenic viruses of laboratory mice. These four pathogens require exclusion from SPF-grade mouse colonies to ensure experimental data reliability.

Ectromelia virus (ECTV), a pathogen belonging to the genus Orthopoxvirus [4], has historically posed a serious threat to laboratory mouse colonies worldwide [5,6]. Mousepox virus (Ectromelia virus) typically enters the host through the skin and replicates near the site of infection. After entering the bloodstream, it infects macrophages in the spleen and liver, leading to necrosis of these organs and severe secondary viraemia [7]. The transmission of mousepox exhibits diverse manifestations with non-specific clinical signs, and the progression of infection is influenced by multiple factors such as viral strain, dosage, route of infection, and host characteristics. Consequently, diagnosis based solely on clinical symptoms is challenging [8].

Mouse hepatitis virus (MHV) is a single-stranded RNA virus belonging to the family Coronaviridae, genus Coronavirus, and is also a common pathogen in laboratory mouse populations [9]. Infection rates have varied by region and era, with reported positivity rates ranging from 1.57% to 12% in North America and Europe [5,6,10]. Most infections are subclinical but can interfere with immunological research [9]. The virus is highly contagious and spreads via the respiratory tract, replicating in the nasal mucosa before disseminating to multiple organs. While adult mice typically experience self-limiting infection, neonates or immunocompromised mice are prone to severe disease and mortality [11,12]. At present, laboratory control measures for MHV rely on strict screening of mouse populations, prevention of wild rodent invasion, and embryo purification.

Mammalian orthoreovirus (MRV) is a double-stranded RNA virus with a capsid composed of inner and outer layers of distinct proteins [13,14]. Based on the characteristics of the σ-1 hemagglutinin protein, MRV is classified into four serotypes: T1L, T2J, T3D, and T4N. Type 3 (Reo-3) is included in health monitoring programs due to its ability to infect both wild and laboratory mice [15,16]. The virus remains prevalent in mouse colonies worldwide, with reported infection rates ranging from 0.02% to 9% in Europe and the United States [5,6,17].

Reo-3 is transmitted via fecal–oral and aerosol routes [18] and can cause severe pathology or fatal encephalitis in young mice [19,20], while adults typically exhibit subclinical infection. The virus is highly resistant in the environment, and contaminated materials can serve as transmission vectors.

Murine parvoviruses (MUV), belonging to the family *Parvoviridae*, are non-enveloped single-stranded DNA viruses with a genome of approximately 5000 nucleotides. Major types include the minute virus of mice (MVM) and the mouse parvovirus (MPV), the latter being one of the most common viruses in laboratory mouse populations. Non-structural proteins (NS-1 and NS-2) are highly conserved among both viruses, whereas the capsid proteins (VP-1, VP-2, VP-3) are more divergent and determine the serogroup [21]. Therefore, in this study, primers were designed based on the conserved region to simultaneously detect both viruses. Seropositivity rates in mouse colonies in Europe and America range from 1% to 10% [21,22], with MPV detected more frequently than MVM. The virus is transmitted orally, replicates in the intestine, spreads to lymphoid tissues, and is shed through fecal excretion, though naturally infected mice exhibit low transmission capability [23]. MPV infection is generally subclinical [24], whereas MVM infection outcomes depend on mouse strain and age [25]. Control strategies primarily include cesarean derivation, embryo transfer, and culling of infected animals, followed by the introduction of virus-free mice [26].

At present, laboratories mainly avoid outbreaks of the four viruses through strict isolation and pathogen detection. However, sensitive, accurate and efficient pathogen detection is essential for disease prevention and control in laboratory mice. Serology is a useful tool for identifying these viruses. However, serological detection has some disadvantages. For example, serology was mostly negative in the initial stages of the disease or in immunodeficient mice, which did not produce a humoral immune response.

Real-time PCR technology demonstrates high sensitivity and specificity through direct detection of pathogen nucleic acids [27]. This study developed a multiplex real-time PCR (mrt-PCR) assay for simultaneous detection of Ectromelia virus (ECTV), murine hepatitis virus (MHV), reovirus type 3 (Reo-3), and murine parvoviruses (MUV). The method enables early infection monitoring, reduces sample consumption and testing costs, and provides effective technical support for the prevention and control of viral infections in laboratory mice.

## 2. Materials and Methods

### 2.1. Clinical Samples and Virus

A total of 16 mice were sourced from research institutions and commercial companies in Beijing, and the heart, liver, spleen, lung, kidney, lymph nodes, reproductive organs, and small intestine were tested, for a total of 128 samples. They were all mice waiting for embryo purification. All tissue samples were collected separately after euthanasia, and samples were stored at −80 °C before processing.

Nucleic acid references of the four target viruses (Ectromelia virus, Murine parvoviruses, Murine hepatitis virus and Reovirus 3 virus) and three non-target viruses (Murine norovirus, Sendai virus, pneumonia virus of mice) were purchased from BeNa Chuanglian Biotechnology, Beijing, China.

### 2.2. Construction of Standard Plasmid

The corresponding gene sequences of each pathogen were retrieved and downloaded from the NCBI database, including: the N gene of MHV (NCBI accession no. NC_048217.1, fragment 29670–31034), the crmD gene of ECTV (NCBI reference sequence NC_004105.1, fragment 5506–6468), the M1 gene of REO-3 (NCBI accession no. NC_077841.1), and the conserved region shared by MPV and MMV (NCBI accession no. NC_001510.1, fragment 2000–2580). Four fragments corresponding to ECTV, MUV, MHV and Reo-3 were separately cloned into the pBluescript II SK+ vector according to the Gene Synthesis Service provided by Sangon Biotech (Shanghai, China). The successful insertion of the target sequences was verified through DNA sequencing conducted by Sangon Biotech (Shanghai, China). The concentration of the constructed standard plasmid was measured using a NanoDrop-2000c spectrophotometer (Thermo Scientific, Waltham, MA, USA), and the plasmid copy number was calculated using the following Equation (1):

(1) $$Copy\;number\left(\frac{copies}{\mu }L\right)=\frac{\left(NA\right)\times \left(\frac{ng}{\mu L}\times 10^{-9}\right)}{DNA\;Length\times MV}$$

Here, NA is Avogadro’s constant, the DNA length is the total base pair count of the cloned sequence plus the vector, and MV is the average molecular weight of a base pair in nucleic acids. After quantification, the standard plasmid solution was stored at −20 °C for subsequent experiments.

### 2.3. RNA In Vitro Transcription for MHV and Reo-III

The universal primers M13 and T7 promoter, as inherent components of the pBluescript II SK+ vector, were used to obtain a product fragment carrying the T7 promoter. The reaction system included 25 μL2× EasyTaq PCR Mix (TransGen Biotech, Beijing, China), 2 μL plasmid solution with a concentration of 1 × 10^6^ copies/μL, forward primers M13-F (5′-GTAAAACGACGGCCAGT-3′) and reverse primer M13-R (5′-CAGGAAACAGCTATGAC-3′) at a final concentration of 0.4 μmol/L, and DEPC-treated water to a total volume of 50 μL. The reaction program was as follows: 95 °C pre-denaturation for 5 s; then 40 cycles containing 94 °C denaturation for 5 s, 55 °C annealing for 15 s, 72 °C extension for 10 s, and a final extension at 72 °C for 30 s.

After 30 min of electrophoresis using 2% agarose gel and 20 V/cm voltage, the PCR product was recovered using FastPure Gel DNA Extraction Mini Kit (Vazyme Biotech, Nanjing, China). Then, RNA in vitro transcription was performed with the PCR product as the target, using the high-yield T7 RNA in vitro transcription kit (Sangon Biotech, Shanghai, China) according to the manufacturer’s instruction manual. The final high-purity RNA was obtained, and its copy number concentration was measured using a NanoDrop 2000, and then stored under −80 °C.

### 2.4. Nucleic Acid Extraction

The viral RNA or DNA from tissue samples was extracted using FastPure Viral DNA/RNA Mini extraction Kit (Vazyme, Nanjing, China, Cat. No. RC313-01), and then was stored at −80 °C.

### 2.5. Design of Specific Primers and Probes

Individual conserved sequences of ECTV, MHV, REO-3, and MUV were aligned using SnapGene pieces (version 6.0.2). Specific primers and probes were designed targeting the crmD region of Ectromelia virus (ECTV), the N region of murine hepatitis virus (MHV), the M1 region of reovirus type 3 (Reo-3), and the vp2 region of Parvoviruses. The specific primers and probes were designed using Primer Premier software (version 3.0.1). We validated the specificity of primers and probes using the Blast tool provided by NCBI, and then further by detecting their mouse susceptible pathogens using quadruple mrt-PCR. The sequence information of the primers and probes is shown in Table 1.

### 2.6. Optimization of the mrt-PCR System

The mrt-PCR reaction system (20 μL) was established according to the manufacturer’s instructions, comprising 10 μL of 2 × mix, 1 μL of E-mix (Vazyme, Nanjing, China), 0.4 μL of each primer (10 μM), resulting in a final primer concentration of 0.2 μM in the reaction system, 0.8 μL of each probe (10 μM), yielding a final probe concentration of 0.05 μM, and 2 μL of template for each pathogen. The remaining volume was supplemented with nuclease-free H_2_O. The amplification parameters for the mrt-PCR system were established as follows: reverse transcription at 55 °C for 5 min, initial denaturation at 95 °C for 10 min, followed by 40 cycles comprising denaturation at 95 °C for 15 s and annealing/extension at 60 °C for 1 min. Upon reaction completion, the system automatically calculated the Ct values.

To optimize the reaction system, we explored different concentrations of primers and probes, as well as varying annealing temperatures.

#### 2.6.1. Optimization of the Concentration of Primers and Probes

This study utilized JMP10 software for experimental design, integrating multi-response criteria with response surface methodology and desirability function analysis for optimization. The central composite face design (CCF) was used to determine the level of the variables and to generate the experimental runs. Eight control factors were selected in the experiment, which were the primer concentration and probe concentration of Ectromelia virus, murine parvoviruses, mouse hepatitis virus, and reovirus type 3. Set three levels for each factor. Specific factors and levels are shown in Table 2.

In this study, the cycle threshold (Ct) values of the four pathogenic plasmids at a concentration of 10^5^ copies/μL were used as four response indicators to evaluate the effect of the mrt-PCR amplification. Based on the response surface methodology, three consecutive runs were performed for each response measurement, and the average was calculated to reduce errors. Data were fitted to a stepwise regression model, and the fit of the stepwise regression model to the observed values was investigated. We applied analysis of variance (ANOVA) to investigate whether the experimental design parameters had a significant effect on the response. Variance tables are also widely used for interactions between factors and the effects of such interactions on the dependent variable [28]. According to the prediction profiler in the JMP software, we predict the maximization willingness, where the CT values of four pathogens were predicted to be close to the minimum. Finally, a confirmation test was conducted. At a plasmid concentration of 10~10^5^ copies/μL for each pathogen, optimized and unoptimized primer-probe concentrations were applied in mRT-PCR assays to examine the potential improvement of detection sensitivity and amplification efficiency of the method by optimized primer-probe concentrations.

#### 2.6.2. Optimizing Annealing Temperature

We established a quadruplex mrt-PCR reaction system following the melting temperatures (TM) of the primers to optimize the annealing temperature. Plasmids (ECTV, MUV) or RNA (REO-3, MHV) templates of each pathogen at the concentration of 10–10^3^ copies/μL, optimization assays were performed at annealing temperatures of 58 °C, 60 °C, and 62 °C, and each assay was carried out in triplicate.

### 2.7. Establishment of a Standard Curve

Using the final reaction conditions and protocol, a quadruple mtr-PCR standard curve was established using a 10-fold serial dilution of a standard plasmid (ECTV, MUV) or RNA (REO-3, MHV) with copy numbers ranging from 1 × 10^6^ to 1 × 10^1^ copies/μL as templates. A linear regression analysis was performed on the Ct values and the logarithm of the plasmid copy numbers. Based on the average Ct values from three replicate measurements obtained through the quadruple mrt-PCR assay, a standard curve was generated for the plasmid. Using the slope of the standard curve, the corresponding amplification efficiency (E) is calculated with the formula: E  =  10^(−1/slope)^ − 1.

### 2.8. Specificity

To evaluate the specificity of the quadruple mrt-PCR assay, reactions were performed using viral RNA or DNA templates from both target and non-target viruses. The non-target viruses included Murine norovirus, Sendai virus, pneumonia virus of mice, Vaccinia virus, Porcine parvovirus, Bovine coronavirus, *Salmonella* spp., *Klebsiella pneumoniae* and *Escherichia coli*. Each virus was set at a concentration of 10^5^ copies/μL in this experiment. Additionally, a negative control consisting of nuclease-free water was included. Each reaction was repeated three times to ensure reliability.

### 2.9. Sensitivity

To evaluate the sensitivity of the quadruple mRT-PCR, standard plasmids (ECTV, MUV) or RNA (REO-3, MHV) were 10-fold serially diluted to a final concentration of 1 × 10^1^ to 1 × 10^7^ copies/μL and used as templates to determine the limit of detection (LOD). Each reaction was repeated three times within a single test. Plasmids with Ct values less than 40 cycles had an amplification efficiency of 80% [29], and the concentration of the plasmids was identified as the lowest detection limit. Although the MIEQ guidelines state that at least 95% probability of the repetitions is commonly used to determine LOD, they also indicate that in practice, this ‘standard’ is variable [30]. In order to ensure true and stable sensitivity in this study, the LOD was set as the minimum concentration of the standard plasmid that yielded positive results in at least 80% of the repetitions.

### 2.10. Repeatability

The mrt-PCR assay’s repeatability (intra-assay) and stability (inter-assay) were measured via coefficients of variation. We serially diluted the standard plasmids or RNA in 10-fold gradients to concentrations ranging from 10^3^ to 10^5^ copies/µL, and performed the detection with at least three replicate wells set up under both intra-assay and inter-assay conditions. CVs were derived from the obtained Ct values. Intra-assay CVs were calculated from replicates within a single run, while inter-assay CVs were derived from replicates across multiple runs. The results showed minimal variability, with both intra-run and inter-run CVs remaining under 3% [31]. This demonstrates the assay’s high reproducibility and consistent performance across experiments.

### 2.11. Clinical Application

To further validate the clinical applicability of the developed mrt-PCR assay, this study collected 128 biological samples. DNA was extracted from the samples using a nucleic acid extraction kit, and the purified DNA was subsequently analyzed by both mrt-PCR and conventional PCR (Detailed procedures are provided in the Supplementary Materials). The sensitivity and accuracy of the mrt-PCR were evaluated for detecting mouse hepatitis virus (MHV), minute virus of mice (MUV), Ectromelia virus (ECTV), and reovirus type 3 (Reo-3) in laboratory mice, with comparisons made to traditional conventional PCR methods.

## 3. Results

### 3.1. Optimization of the Quadruple mrt-PCR Reaction System

#### 3.1.1. Optimization of Primer and Probe Concentrations

We determined the experimental scheme for optimizing primer and probe concentrations using JMP software. For each response measurement, three consecutive runs were performed to obtain averaged values for error reduction. The acquired data were fitted to a stepwise regression model, and we analyzed the goodness of fit of the stepwise regression model to the observed values. The goodness-of-fit summary for each response regression model is shown in Table 3.

The goodness-of-fit of the models was evaluated using R^2^ and adjusted R^2^. The results demonstrated that all models exhibited satisfactory explanatory power for the observed data. Particularly, the minute virus CT model achieved an adjusted R^2^ of 0.503, indicating its regression equation had relatively superior fitting performance. Notably, the adjusted R^2^ values were consistently lower than the R^2^ values by an average of 0.17 across all models, suggesting potential overfitting issues. Therefore, variable selection methods should be implemented to further optimize model accuracy.

Analysis of prediction errors through root mean square error (RMSE) demonstrated that while the MHV, Reo-3 and MUV CT models maintained high precision with RMSE ranging between 0.26–0.30 and relative errors controlled within 0.9–1.1%, the ECTV CT model exhibited significantly higher RMSE (0.428), representing a 64% increase compared to other models, which may be associated with experimental conditions or other technical factors affecting its quantification accuracy.

We performed ANOVA on the regression model to assess the joint significance of all independent variables on the response using F-tests [28]. The F-test evaluates whether the overall regression coefficients differ significantly from zero (*p* < 0.05 indicates a significant collective impact of predictors on the dependent variable). Table 4 is the standard ANOVA output:

The F-ratio is computed as: $\;F=\frac{Mean\;Square\;(Error)}{Mean\;quare\;(Model)}$. This metric quantifies whether the variation explained by the model (signal) is significantly greater than random error (noise). A higher F-ratio indicates stronger evidence that the independent variables significantly influence the response variable. The statistical analysis demonstrates that the model for Minute Virus of Mice (MUV) CT values shows the strongest effect (F = 5.90), while Hepatitis Virus CT values exhibit the weakest yet still significant effect (F = 3.13). All models yield highly significant *p*-values (*p* < 0.0001); this collectively indicates that the experimental design’s independent variable combinations significantly influence all CT value categories.

We conducted lack-of-fit tests to systematically analyze the sources of unexplained variation in the models. Table 5 presents the lack-of-fit test results for each regression model.

The table systematically presents the lack-of-fit test results for four viral CT value models. All models demonstrated statistically acceptable fit (*p* > 0.05: MHV 0.34, ECTV 0.42, Reo-3 0.26, MUV 0.12), with the MUV model showing superior goodness-of-fit (Max R^2^ = 0.9555), albeit with the highest lack-of-fit to pure error ratio (1.7703), suggesting potential unmodeled nonlinearity. The ECTV model exhibited the largest error terms (lack-of-fit MS = 0.187519) but the most stable F-ratio (1.1490). With consistent pure error degrees of freedom (12–14) across models ensuring reliable error estimation, the higher lack-of-fit DF values (58/56) for MHV and Reo-3 models indicate potential benefits from increased sample size to enhance model robustness in future studies.

Based on the prediction profiler in JMP software, we performed optimization to minimize the CT values for MHV, ECTV, Reo-3, and MUV amplification. The optimization results indicate that the optimal primer concentrations for achieving minimal CT values are as shown in Table 6.

We conducted validation experiments to evaluate the performance of optimized versus unoptimized primer-probe concentrations in mrt-QCR detection. Serial dilutions of plasmid or RNA standards (10–10^5^ copies/μL) for each pathogen were analyzed using both concentration sets to systematically assess the improvements in detection sensitivity and amplification efficiency. All reactions were performed in triplicate, with quantitative results expressed as mean values. Results are shown in Table 7.

The comparative analysis revealed minimal differences in CT values between unoptimized and optimized primer/probe concentrations across all four viral templates at higher sample concentrations (E2–E5 copies). However, at the low-concentration E1 copy level, only the optimized system successfully detected the templates, demonstrating significantly enhanced detection sensitivity.

#### 3.1.2. Annealing Temperature Optimization

**Figure 1:**
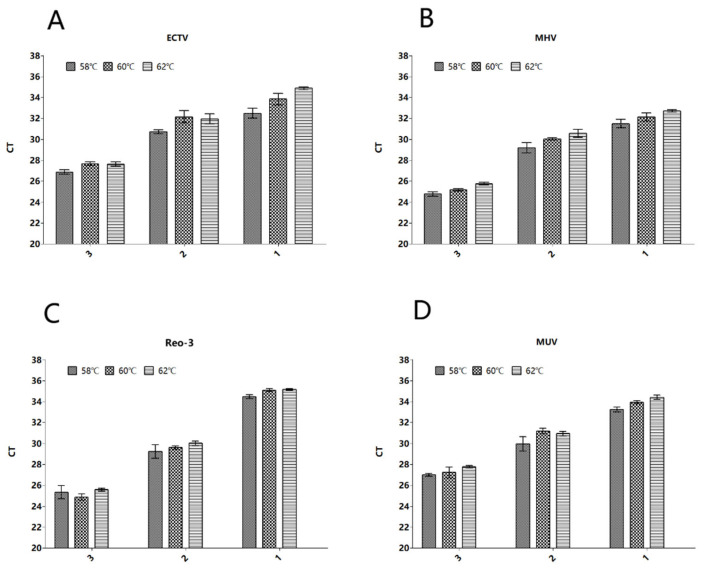
Optimization results of denaturation temperature of (**A**) ECTV, (**B**) MHV, (**C**) Reo-3, and (**D**) MUV. 1—the result with the denaturation temperature of 62 °C. 2—the result with the denaturation temperature of 60 °C. 3—the result with the denaturation temperature of 58 °C.

When the annealing temperature is 58 °C, the Ct values of different pathogens are relatively low, thus confirming 58 °C as the optimal annealing temperature (Figure 1).

#### 3.1.3. Final Reaction Setup for Quadruplex mrt-PCR

The mrt-PCR reaction conditions were set as follows: initial denaturation at 95 °C for 3 min, followed by 40 cycles consisting of reverse transcription at 55 °C for 5 min (for RNA templates), denaturation at 95 °C for 10 s, annealing at 95 °C for 10 s, extension at 58 °C for 1 min with fluorescence acquisition during this step.

### 3.2. Standard Curve

**Figure 2:**
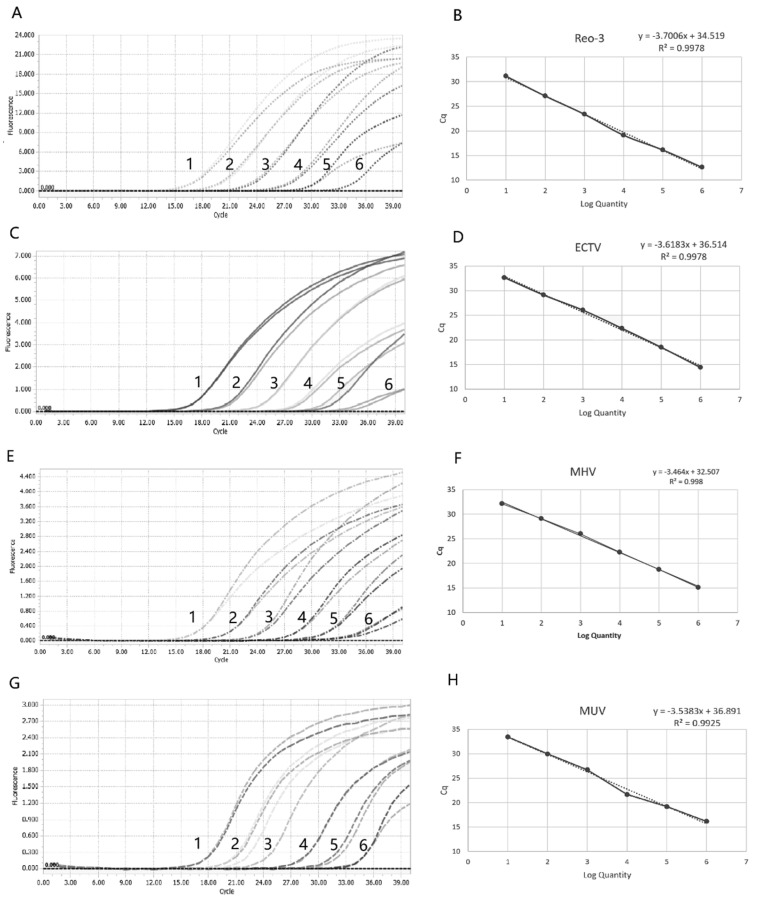
Amplification and standard curves of Reo-3, ECTV, MHV, and MUV. (**A**) Amplification curve of Reo-3. (**B**) Standard curve of mrt-PCR for Reo-3. (**C**) Amplification curve of ECTV. The curves 1–6: standard plasmid or RNA concentrations from 1 × 10^8^ to 1 ×10^2^ copies/μL. (**D**) Standard curve of mrt-PCR for ECTV. (**E**) Amplification curve of MHV. (**F**) Standard curve of mrt-PCR for MHV. (**G**) Amplification curve of MUV. (**H**) Standard curve of mrt-PCR for MUV. The curves 1–6: standard plasmid or RNA concentrations from 10^8^ to 10^2^ copies/μL.

To establish the standard curves for quadruplex mrt-PCR, a 10-fold serial dilution of standard plasmid or RNA was prepared using template concentrations ranging from 1 × 10^8^ copies/μL to 1 × 10^2^ copies/μL. The resulting standard curves demonstrated excellent amplification efficiency and high correlation coefficients, validating the analytical reliability. The obtained parameters were as follows: for Reo-3, R^2^ =0.9978 with efficiency (E) = 86.30%; for ECTV, R^2^ =0.9978 with E = 88.96%; for MUV, R^2^ =0.9925 with E = 91.69% (Figure 2); and for MHV, R^2^ =0.9925 with E = 94.39%. These high R^2^ values indicate strong linearity between the logarithm of copy numbers and corresponding CT values, confirming that the plasmid standards enable reliable quantification across the specified concentration range.

### 3.3. Specificity Analysis

**Figure 3:**
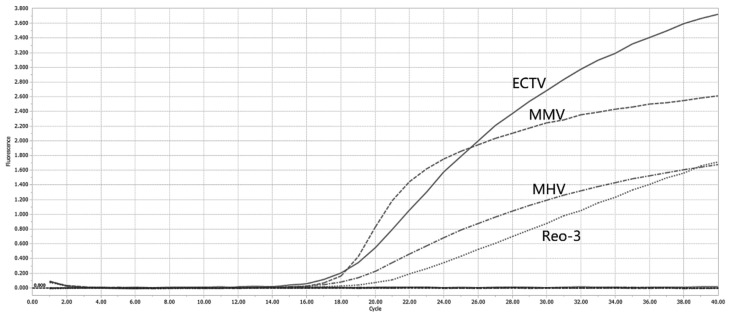
The result of the specificity analysis. The four lines represent MHV, ECTV, Reo-3, and MUV, respectively.

To evaluate the specificity of the assay, mrt-PCR was performed to amplify nucleic acids from MHV, ECTV, Reo-3, MUV, and other common murine viruses (Murine norovirus, Sendai virus, pneumonia virus of mice). The results demonstrated positive detection exclusively for MHV, ECTV, Reo-3, and MUV, while all other viral nucleic acid samples tested negative (Figure 3). These findings confirm that the quadruplex detection method for MHV, ECTV, Reo-3, and MUV exhibits excellent specificity without cross-reactivity to non-target murine pathogens.

### 3.4. Sensitivity Analysis

To determine the limit of detection (LOD) of the mrt-PCR assay, a tenfold dilution series of standard plasmid (ranging from 1.08 × 10^9^ to 1.08 × 10^0^ copies/μL) was tested three times. The assay exhibited exceptional sensitivity, consistently detecting all four target viruses at concentrations as low as 1.08 copies/μL of standardized plasmid DNA. Further validation using plasmid templates (1.08 × 10^3^ to 1.08 × 10^0^ copies/μL) in 24 replicate tests established Ct = 40 as the positivity threshold (Ct ≤ 40: positive; Ct > 40 or undetected: negative) [29]. The assay achieved 100% detection rate at concentrations between 1.08 × 10^3^ and 1.08 × 10^1^ copies/μL. At the single-copy level (1.08 × 10^0^ copies/μL), the detection results were as follows (Table 8). Therefore, the limits of detection (LOD) for ECTV, MHV, Reo-3, and MUV were determined to be 1.08 × 10^1^ copies/μL, 1.14 × 10^1^ copies/μL, 2.38 ×10^1^ copies/μL, and 1.08 × 10^1^ copies/μL, respectively.

### 3.5. Repeatability Analysis

To evaluate the repeatability of the newly developed mrt-PCR assay, plasmid DNA templates of MHV, ECTV, Reo-3, and MUV at three concentrations (1.08 × 10^5^, 1.08 × 10^4^, and 1.08 × 10^3^ copies/μL) were amplified and quantified. The results demonstrated excellent intra-assay precision, with coefficient of variation (CV) values < 0.5% across all targets and concentrations, indicating highly consistent performance within the same run. For inter-assay repeatability, CV values ranged between 0.38–2.28%, showing slightly reduced but still exceptional reproducibility across different experimental batches. Notably, all CV values remained well below the 3% threshold [31], confirming the assay’s robust and reliable performance under varying testing conditions. This level of precision meets or exceeds the performance criteria recommended by the MIQE guidelines for diagnostic mrt-PCR assays (Table 9).

### 3.6. Diagnostic Validation with Clinical Samples

To further validate the clinical applicability of the developed mrt-PCR assay, 128 murine tissue samples from 16 mice were tested using mrt-PCR, and the results were compared with those obtained by conventional PCR. Among the 128 samples, mrt-PCR detected 28 positive for Ectromelia virus (ECTV), with a detection rate of 21.88%; 18 positive for mouse hepatitis virus (MHV), with a detection rate of 14.06%; 10 positive for reovirus type 3 (Reo-3), with a detection rate of 7.81%; and 12 positive for minute virus of mice (MUV), with a detection rate of 9.38% (Table 10).

These samples were also analyzed using conventional PCR. The conventional PCR results showed 15 samples positive for ECTV (10.16%); 1 sample positive for MHV (0.78%); 5 positive samples for Reo-3 (3.91%); and 6 positive samples for MVM (4.69%) (Table 10).

Notably, the discordant samples between the two methods exhibited high Ct values (e.g., 35.12), indicating low viral loads. This suggests that the mrt-PCR assay is more sensitive than conventional PCR in detecting low quantities of viral RNA. For ECTV, MHV, Reo-3, and MUV, the results of the mrt-PCR and conventional RT-PCR were consistent, further demonstrating the accuracy and reliability of the multiplex detection system.

## 4. Discussion

Currently, in the field of molecular biology, the primary detection methods for mousepox (ECTV), mouse hepatitis virus (MHV), reovirus type 3 (Reo-3), and mice minute virus (MUV) include conventional bacterial culture, PCR, nested PCR, and qPCR techniques, in which qPCR technology effectively offers superior sensitivity and specificity compared to the other methods. Notably, mrt-PCR enables simultaneous quantitative detection of multiple target genes from different pathogens in a single-tube reaction, significantly enhancing efficiency and practicality [28].

Currently, the experimental approaches for optimizing primer-probe concentrations in mrt-PCR detection kits remain relatively limited and underdeveloped. Most commercially available mrt-PCR kits lack systematic optimization of primer-probe concentrations tailored for different pathogens, typically employing uniform concentrations across all targets without considering the variations in amplification efficiency, secondary structure formation, or potential interference between different primer-probe sets [29].

This standardization gap often results in suboptimal assay performance, including imbalanced amplification of targets, reduced sensitivity for certain pathogens, and increased susceptibility to false-negative results, particularly when detecting low-abundance targets in complex clinical or environmental samples. The development of robust, pathogen-specific concentration optimization protocols represents a critical unmet need in mrt-PCR technology advancement.

This study employed a statistically rigorous response surface methodology (RSM) approach, utilizing design of experiments (DOE) principles to systematically investigate the effects of varying primer-probe concentrations for different pathogens on mrt-PCR amplification performance. The experimental design incorporated eight critical factors (independent variables) comprising the concentration parameters for primers and probes targeting all four viruses, with four key response variables (output parameters) defined as the CT values for each pathogen-specific mrt-PCR reaction. This methodological framework enables the identification of optimal combination conditions that simultaneously influence all four response variables. RSM, traditionally applied in industrial process optimization, product development, and quality improvement initiatives [30], was adapted here to establish quantitative relationships between response variables and multiple input factors, with the primary objective of optimizing these influential parameters through minimal experimental iterations while maintaining statistical power, as a systematic experimental optimization strategy. The core principle of RSM involves using mathematical models to analyze the combined effects of multiple factors (such as primer and probe concentrations) on response variables (e.g., Ct values of four viruses), thereby rapidly identifying the optimal combination of conditions. This method significantly outperforms traditional univariate optimization approaches and is particularly suitable for screening conditions in complex systems, such as multiplex qPCR assays.

According to the DOE methodology, an optimized combination of the primer-probe concentration was achieved, which was further confirmed by a comparative experiment between the optimized concentrations and the non-optimized ones. As the results show, the DOE-optimized primer-probe combinations exhibited significant improvements, including an enhancement in detection sensitivity, and maintained amplification efficiency within optimal ranges. In this study, model goodness-of-fit was evaluated using R^2^ and adjusted R^2^. The results indicated that all models exhibited satisfactory explanatory power for the observed data, with the adjusted R^2^ for the minute virus Ct value model reaching the highest value (0.503). Notably, the adjusted R^2^ values were consistently lower than the corresponding R^2^ values across all models, with a mean difference of 0.17, suggesting a potential risk of overfitting. In stepwise regression analysis, the introduction of interaction terms or nonlinear effects did not significantly improve these values, thereby ruling out inappropriate model selection as a contributing factor. We attribute the suboptimal R^2^ values primarily to experimental error. All liquid handling procedures in this study were performed manually, and experimental error was inevitably amplified under conditions of low and narrow concentration ranges for primers (0.2–0.6 μM) and probes (0.02–0.08 μM). Replacing manual liquid handling with automated liquid handling can effectively reduce such errors. The limited throughput of the qPCR instrument resulted in unavoidable inter-group variation when the number of experimental groups was large; this issue can be ameliorated by increasing the number of replicate measurements. Furthermore, the use of qPCR instruments with higher single-run sample capacity (e.g., 384-well plates) can also effectively reduce inter-group variation. Additionally, it is undeniable that alternative statistical software or modeling approaches may better address the problem of overfitting compared with those employed in the present study.

Analysis of prediction error based on root-mean-square error (RMSE) demonstrated that the Ct value models for mouse hepatitis virus (MHV), reovirus type 3 (Reo-3), and minute virus (MUV) retained high predictive precision, with RMSE values ranging from 0.26 to 0.30 and relative errors constrained to 0.9–1.1%. By contrast, the RMSE of the Ct value model for Ectromelia virus (ECTV) was significantly increased to 0.428, a 64% rise relative to the other models, which may be attributable to the specificity of the ECTV primer–probe set. In multiplex detection platforms, non-specific primer binding constitutes the predominant factor impairing the amplification efficiency of target sequences. Such interference arises from two principal sources: cross-interactions between primers designed for distinct pathogens, and non-specific binding of primers to heterologous pathogen templates. In the present study, ECTV primers were disproportionately affected by such non-specific interactions, which plausibly underlie the elevated variance observed for this target. Most widely used primer–probe design software currently evaluates non-specific binding only between primer pairs and between primers and their intended target sequences. Future development and adoption of dedicated analytical tools for the assessment of inter-pathogen primer–primer and primer–template cross-binding in multiplex assays would effectively eliminate the detrimental effects of such non-specific interactions on detection performance.

To optimize our multiplex mrt-PCR assays, we evaluated the detection methods for ECTV, MHV, Reo-3, and MUV by measuring analytical sensitivity and specificity. Each pathogen demonstrated a detection limit of 1 ×10^1^ copies/μL, with both intra-assay and inter-assay coefficients of variation below 2%. The established assays exhibited excellent reproducibility and sensitivity, providing a rapid, sensitive, and specific diagnostic tool for laboratory mouse pathogens, including MHV, ECTV, Reo-3, and MUV.

Standard curves generated from singleplex and mrt-PCR assays showed R^2^ values of 0.99–1 for each artificial template, with amplification efficiencies (E) falling within recommended ranges (90–110% for singleplex and 80–120% for multiplex assays) [31]. Importantly, our mrt-PCR assay demonstrated no interference from the presence of other primers and probes when compared to corresponding single-plex detections.

## 5. Conclusions

This study developed an MRT-PCR assay for the simultaneous detection and differentiation of MHV, ECTV, Reo-3, and MUV. With its demonstrated specificity, sensitivity, and efficiency, the developed mrt-PCR assay represents a promising tool for clinical diagnosis and epidemiological studies of these pathogens in laboratory mouse colonies and related biological products.

## Figures and Tables

**Table 1:** Sequences of primers and probes.

Pathogens	Name	Sequence (5′-3′)	Fragment Length	Melting Temperature (Tm)
MHV	F	TATTGCCTCAGGGCTTTTATGTT	118 bp	60.3
	R	CGCTGGTTGGAACTGCTTCT		60.3
	P	(VIC)TGCTAGCCGATCTGGTTCG(MGB)		
Reo-3	F	GTGCGCCAAGACTGGTTATAG	120 bp	57.8
	R	CAGCCTTAGCAGATACCCTCC		58.4
	P	(Texred)TTCGCCTACGACAAGC(MGB)		
ECTV	F	AGCCTGCAACCGTTTGATCC	118 bp	62.3
	R	TCATAGTCCCGCGTGTCTAAGTT		60.8
	P	(FAM) TTCGCAGTAGTATCCG (MGB)		
MUV	F	GGTGCGGAACCGTTGAAGA	117 bp	61.4
	R	CCACCAACCAACCATCCCTTA		61.9
	P	(CY5)CGCCATCGTACCTTAGTCC(MGB)		

Note: F—forward primer, R—reverse primer, P—probe. The melting temperatures (TM) were obtained from predictions generated by Primer5 software.

**Table 2:** Control factors and levels.

Factor	Unit	Level		
		1	2	3
MUV primer	μM/L	0.2	0.4	0.6
Reo-3 primer	μM/L	0.2	0.4	0.6
ECTV primer	μM/L	0.2	0.4	0.6
MHV primer	μM/L	0.2	0.4	0.6
MUV probe	μM/L	0.02	0.05	0.08
Reo-3 probe	μM/L	0.02	0.05	0.08
ECTV probe	μM/L	0.02	0.05	0.08
MHV probe	μM/L	0.02	0.05	0.08

**Table 3:** The goodness-of-fit summary for each response regression model.

Source	R-Squared	Adjusted R-Squared	Root Mean Square Error (RMSE)	Observations
MHV CT	0.53	0.38	0.26	93.00
ECTV CT	0.50	0.33	0.43	93.00
Reo-3 CT	0.50	0.35	0.28	93.00
MUV CT	0.65	0.50	0.31	93.00

**Table 4:** ANOVA table.

Source		Degrees of Freedom (DF)	Sum of Squares (SS)	Mean Square (MS)	F-Ratio	*p*-Value
MHV CT	Model	22	5.28	0.24	3.53	<0.0001 *
	Error	70	4.76	0.07		
	Total	92	10.04			
ECTV CT	Model	24	12.56	0.52	2.86	0.0004 *
	Error	68	12.46	0.18		
	Total	92	25.02			
Reo-3 CT	Model	22	9.33	0.42	3.22	<0.0001 *
	Error	70	9.23	0.13		
	Total	92	18.56			
MUV CT	Model	19	9.22	0.49	5.90	<0.0001 *
	Error	73	6.01	0.08		
	Total	92	15.23			

Note: *—statistically significant at the significance level α = 0.05.

**Table 5:** The lack-of-fit test results for each regression model.

Source		Degrees of Freedom (DF)	Sum of Squares (SS)	Mean Square (MS)	F-Ratio	*p*-Value	Max R^2^
MHV CT	Lack-of-fit	58	4.09	0.07	1.26	0.34	0.93
	Pure Error	12	0.67	0.06			
	Total Error	70	4.76				
ECTV CT	Lack-of-fit	56	10.50	0.19	1.15	0.42	0.92
	Pure Error	12	1.96	0.16			
	Total Error	68	12.46				
Reo-3 CT	Lack-of-fit	56	7.82	0.14	1.40	0.26	0.92
	Pure Error	14	1.41	0.10			
	Total Error	70	9.23				
MUV CT	Lack-of-fit	54	4.63	0.09	1.77	0.12	0.96
	Pure Error	14	0.68	0.05			
	Total Error	68	5.31				

**Table 6:** Optimal concentrations of primers and probes for each pathogen.

	MHV Primer	Reo-3 Primer	ECTV Primer	MUV Primer	MHV Probe	Reo-3 Probe	ECTV Probe	MUV Probe
Concentration (μmol/L)	0.2	0.6	0.6	0.44	0.08	0.06	0.08	0.08

**Table 7:** Comparison of optimized performance results.

Copy Number	Unoptimized Primer/Probe Concentrations				Optimized Primer/Probe Concentrations			
	ECTV	MHV	Reo-3	MUV	ECTV	MHV	Reo-3	MUV
10^5^	21.26	22.44	17.07	21.81	20.82	22.35	17.25	21.23
10^4^	24.32	25.38	20.77	24.42	24.48	25.17	21.14	24.10
10^3^	27.12	28.83	23.66	27.81	26.07	27.52	23.41	27.20
10^2^	31.70	30.53	28.74	32.17	28.53	30.10	27.52	29.08
10	Un *	Un	Un	Un	30.54	31.17	31.22	30.52

Note: * Un—Undetected.

**Table 8:** Limit of detection (LOD) assessment for the mrt-PCR assay.

Pathogen	Copies/μL	Replicates	Positive Detections	Detection Rate	≥80% Threshold
ECTV	1.08	24	11	54.16	no
MHV	1.14	24	8	33.3	no
Reo-3	2.38	24	9	37.5	no
MUV	1.08	24	11	54.16	no

**Table 9:** Intra- and inter-assay variability of the mrt-PCR assay.

Pathogen	Concentration (Copies/µL)	Intra-Assay		Inter-Assay	
		Ct Mean ± SD	CV (%)	Ct Mean ± SD	CV (%)
ECTV	10^5^	19.57 ± 0.08	0.39%	19.15 ± 0.44	2.28%
	10^4^	23.54 ± 0.08	0.32%	23.23 ± 0.38	1.64%
	10^3^	27.54 ± 0.04	0.15%	27.05 ± 0.50	1.84%
MHV	10^5^	19.40 ± 0.35	1.80%	18.98 ± 0.2	1.04%
	10^4^	22.81 ± 0.28	1.21%	22.63 ± 0.25	1.11%
	10^3^	26.40 ± 1.35	1.34%	26.39 ± 0.35	1.34%
Reo-3	10^5^	18.27 ± 0.04	0.21%	18.05 ± 0.18	0.97%
	10^4^	22.92 ± 0.07	0.31%	22.52 ± 0.4	1.78%
	10^3^	25.97 ± 0.15	0.59%	25.94 ± 0.06	0.23%
MUV	10^5^	19.61 ± 0.04	0.22%	19.68 ± 0.08	0.38%
	10^4^	24.16 ± 0.12	0.50%	23.85 ± 0.42	1.77%
	10^3^	27.27 ± 0.10	0.35%	26.66 ± 0.49	1.85%

**Table 10:** Detection of clinical samples.

Methods	Pathogen	Positive/Total Number	Positive Rate
mrt-PCR	ECTV	28	21.88%
	MHV	18	14.06%
	Reo-3	10	7.81%
	MUV	12	9.38%
conventional PCR	ECTV	15	10.16%
	MHV	1	0.78%
	Reo-3	5	3.91%
	MUV	6	4.69%

## Data Availability

The original contributions presented in this study are included in this article. Further inquiries can be directed to the corresponding author.

## Supplementary Materials

The following supporting information can be downloaded at: https://www.mdpi.com/article/10.3390/vetsci13030217/s1, Table S1: The Primer sequence of traditional conventional PCR method.
