# Supplementary material for: Multiplex One-Step qPCR/RT-qPCR Assays for Detection of Ectromelia Virus, Murine Hepatitis Virus, Reovirus Type 3, and Parvoviruses
Source: Vet Sci. 2026 Feb 25;13(3):217. doi: 10.3390/vetsci13030217 (PMC13029953; doi:10.3390/vetsci13030217)
Supplement: Supplementary file 1 [file vetsci-13-00217-s001.zip › vetsci-4109449-supplementary.pdf]

Supplementary Materials：Traditional conventional PCR method

1. Traditional conventional PCR

In this study, we also established a common PCR method to detect four pathogens. The relevant primers are shown in Table S1.

Table S1. The Primer sequence of traditional conventional PCR method.

Pathogens	Name	Sequence (5′-3′)
ECTV	ECTV500-3F	CGTCGTGGGTGTTAGTTGTCTAG
	ECTV500-3R	GAGGCAAATGTAGCAGTAATCAAG
MHV	MHV300-1F	AATACCCGCTCTGATATTGTCG
	MHV300-1R	GGCACTTTGCTTCGTTACTTG
Reo-3	Roe400-3F	ATTGCTACCCCGAAATGTTTT
	Roe400-3R	ACCAATCTGTGAGCATTCCATC
MUV	MPMV200-1F	TGAACTTGGACTAAGGTACGATGG
	MPMV200-1R	AGCGGCGTCAGATGGATT

2. PCR procedure

The reaction system included 10 μL 2× Accur START One Step RT-PCR Kit Dye Plus (Vazyme International LLC.), 2 μL target solution, each primers at a final concentration of 0.2 μmol/L, adding DEPC-treated water to a total volume of 20 μL. The reaction program was as follows: reverse transcription in 55℃ for 5 min, 95℃ pre-denaturation for 3 min; then 40 cycles containing 95℃ denaturation for 15 s, 64℃ annealing for 20 s; 72℃ extension for 20 s.

3. Electrophoresis

30 minutes of electrophoresis was performed using 2% agarose gel and 20 V/cm voltage.
